# Intense isolated attosecond pulses from two-color few-cycle laser driven relativistic surface plasma

**DOI:** 10.1038/s41598-022-17762-3

**Published:** 2022-08-11

**Authors:** Sudipta Mondal, Mojtaba Shirozhan, Shivani Choudhary, Kwinten Nelissen, Paraskevas Tzallas, Dimitris Charalambidis, Katalin Varjú, Subhendu Kahaly

**Affiliations:** 1 ELI-ALPS, ELI-HU Non-Profit Ltd., Wolfgang Sandner utca 3., Szeged, 6728 Hungary; 2 Foundation for Research and Technology-Hellas, Institute of Electronic Structure & Laser, 70013 Heraklion (Crete), Greece; 3 Department of Physics, University of Crete, PO Box 2208, 71003 Heraklion (Crete), Greece; 4 Institute of Physics, University of Szeged, Dóm tér 9, Szeged, 6720 Hungary; 5 Department of Optics and Quantum Electronics, University of Szeged, Dóm Tér 9, Szeged, 6720 Hungary

**Keywords:** Optical physics, Plasma physics, High-field lasers

## Abstract

Ultrafast plasma dynamics play a pivotal role in the relativistic high harmonic generation, a phenomenon that can give rise to intense light fields of attosecond duration. Controlling such plasma dynamics holds key to optimize the relevant sub-cycle processes in the high-intensity regime. Here, we demonstrate that the optimal coherent combination of two intense ultrashort pulses centered at two-colors (fundamental frequency, $\omega$ and second harmonic, $2\omega$) can lead to an optimal shape in relativistic intensity driver field that yields such an extraordinarily sensitive control. Conducting a series of two-dimensional (2D) relativistic particle-in-cell (PIC) simulations carried out for currently achievable laser parameters and realistic experimental conditions, we demonstrate that an appropriate combination of $\omega -2\omega$ along with a precise delay control can lead to more than three times enhancement in the resulting high harmonic flux. Finally, the two-color multi-cycle field synthesized with appropriate delay and polarization can all-optically suppress several attosecond bursts while favourably allowing one burst to occur, leading to the generation of intense isolated attosecond pulses without the need of any sophisticated gating techniques.

## Introduction

Intense sub-femtosecond pulses of X-ray or extreme ultra-violet (XUV) radiation are of primary interest, in several areas of fundamental and applied research including plasma physics, material science, chemical-biology, etc., driving the quest for the new generation of bright light sources spanning the aforementioned spectral range. A coherent attosecond waveform, the shortest of such fields, is produced primarily via the high harmonic generation (HHG) process^1,2^ during the interaction of high-intensity short-pulse lasers with gaseous media^3^, ablated plasma plumes^4^, semiconductor crystals^5^, and surface plasmas^6,7^. A common theme in all these techniques is the optimal control of ultrafast electron dynamics for efficient and tunable HHG, currently being utilized in the attosecond beamlines existing in academic laboratories as well as large-scale research facilities^8–11^. One such control is to use multi-color pulses to shape the laser field that is driving the interaction. In the relativistic domain, such control is achieved by utilizing an appropriately synthesized light field^12^ and through a proper choice of interaction media^13^.

When a high-power light field is tightly focused on an overdense plasma surface, the intensity reaches a value such that electron motion becomes relativistic over sub-cycle timescale, launching ultrafast charge separation dynamics that is responsible for the subsequent emission of coherent XUV radiation^6,7^. Such a source has demonstrated its scalability with incident laser intensity^14^ and showed its potential in terms of efficiency^14,15^, spectral extent^16,17^, beam properties^18–20^, divergence control^13,21,22^, pulse energy^23,24^, *attosecond* pulse duration^25,26^ and pulse separation^17,27,28^. In addition, a successful combination of the damage-less nature of the medium, relativistic optics,^22^ and efficient coherent frequency up-conversion together hold promise to offer the only potential approach for achieving super high intensities^26,29,30^ needed for probing vacuum quantum electrodynamics with currently available high power lasers^31–34^. Thus, the importance of boosting the quality of surface plasma based sources^8^ is of central importance.

Relativistic high harmonics from plasma mirrors offer a promising path for generating bright attosecond light pulses. However, a major challenge is to tailor the process to generate isolated attosecond pulses, well adapted to pursue timed-resolved investigations. Different techniques for generating isolated attosecond pulses from solid density plasma have been proposed and theoretically explored. Each one of them has its relative advantages and is applicable under different pulse parameter regimes. Techniques based on the controlled introduction of the spatio-temporal coupling^35^, utilization of polarization gating^36,37^ carrier envelope phase control in few-cycle pulses^28,38^ have been proposed and explored. In two-color driven surface plasma, some initial one-dimensional simulations and models^39,40^ hinted at the possibility of improved high harmonic efficiency. Recently, two experiments demonstrated the potential of two-color driven sub-cycle control of plasma mirror in the moderately relativistic case^12,41^.

In this study, we undertake a series of two-dimensional (2D) relativistic particle-in-cell simulations, mimicking realistic experimental conditions, and investigate two-color optimal coherent control of ultrafast relativistic dynamics of a solid density surface plasma and the subsequent generation of high order harmonics. Such a multicolor drive field is currently accessible. Experimentally, multiple options have been demonstrated and utilized to generate co-linear two-color ultrashort pulses. Waveform shaping on sub-cycle time scales is achieved through the usage of waveform synthesizers^42–45^ or alternatively through the phase control of each frequency component in frequency space^46–48^. Another scheme that has been used quite frequently to generate the bicolor field from the fundamental near the interaction point, while allowing quasi-independent control over the two. In this case, the functional building blocks of the optical setup consist of frequency doubling, independent polarization control, and timing control that generates two-color fields in the colinear mode of operation^12,41,49^. Our computational results demonstrate that by mixing a sufficient amount of second harmonic (2$\omega$) to the fundamental driving pulse collinearly and tuning the relative delay between the main pulse ($\omega$) and its second harmonic ($2\omega$) precisely, one can effectively control the surface plasma oscillations to optimize high harmonic generation process and can subsequently reach a regime where isolated attosecond bursts can be produced.

## The surface plasma high harmonic generation

The wide range of accessible laser-matter parameter space and a diversity of allowed experimental geometries permit several mechanisms to concomitantly participate in the high harmonic generation process^50,51^. Two key parameters that influence the interaction regime^6,7,13^ are the dimensionless peak laser vector potential $a_0=8.53 \times 10^{-10}\times (I_{Wcm^{-2}}\lambda _{\mu m}^2)^{1/2}$ (where *I* is the incident laser intensity and $\lambda$ is the carrier wavelength of incident light and subscripts indicate the respective units in the expression) and the plasma density gradient scale length $L= |n_{e}/\nabla n_{e}|_{n_{e}=n_{c}}$ ($n_{e}$ being the plasma electron density and $n_{c}$ is the critical density at fundamental laser frequency). Several experiments have been conducted accessing the different approaches to generate and optimize HHG from surface plasma in the relativistic regime of interaction in order to enhance the effectiveness of the generation process (for a comprehensive summary, see^2,51^ and the references therein).

When a high contrast, relativistically intense ($a_0 \ge 1$) ultrashort laser pulse impinges upon an optically flat solid density surface, the rising front of the laser pulse ionizes the surface material and generates a nearly step-like plasma vacuum interface. The remaining part of the pulse interacts with the generated plasma mirror, resulting in periodic sub-cycle relativistic motion of the surface electrons. Depending on the nature of the relativistic collective dynamics of such surface electrons, different processes can take place. Under certain conditions, such a process imprints sub-cycle periodic phase modulations in the temporal profile of the reflected pulse, leading to high harmonic generation in the frequency domain. This process is known as the relativistic oscillating mirror (ROM) mechanism^6,14,22,52^. However, under a different set of interaction conditions, a more favourable bunching of the electron can take place, undergoing collective acceleration in synchrotron-like trajectories. The relativistic electron bunch and the ensuing ultrafast nanometer-scale dynamics lead to the generation of synchrotron-like coherent radiation called coherent synchrotron emission (CSE)^53–56^. The emitted high harmonics in CSE have distinct spectral characteristics^51,57^ and temporal features^58^ compared to ROM harmonics, and both the processes have been identified in experiments^16,28,54,59^.

The physical picture of the interaction of a relativistic intensity laser pulse with a step-like plasma vacuum interface as reconstructed from the experimental observations and PIC simulations is described below. The surface electrons are pushed into the target by the initial part of the optical cycle (push phase). This push-phase usually takes place during the initial half of the optical cycle. In the latter half of the optical cycle, a restoring force is developed due to charge separation. The electrons are pulled back into the vacuum by the combined effect of the optical field and the field generated by the charge separation and generate a relativistic energy electron bunch (pull phase)^22,53,60,61^. This push-pull dynamics continues in each optical cycle of the laser pulse and plays a significant role in high harmonic generation. Although the duration of complete electron dynamics in relativistic laser-plasma interactions is much longer and also generates radiation bursts in other wavelengths^62^, however, electron dynamics that generate attosecond bursts occur in sub-cycle time duration^7^.

In order to probe the process in the relevant intensity and pulse duration regime, we undertake a series of virtual experiments by conducting two-dimensional (2D) relativistic Particle-in-Cell (PIC) simulations using the code PICCANTE^63^. An 800 nm, p-polarized Gaussian pulse (Gaussian profile in space and $\cos ^2$ profile in time) with the dimensionless peak laser vector potential $a_0 = 10$ and pulse-width $\mathrm {\sim 8}$ fs [Full-Width-at-Half-Maxima (FWHM) of intensity] is incident on a plane target at an angle 50$^\circ$. The focal spot diameter ($2w_0$) is set to 5 $\mathrm {\upmu m}$. We emulate fully ionized semi-infinite carbon plasma with initial peak electron density $n_{0} = 50 n_{c}$ and exponential plasma density profile $n_{e}(x)=\frac{n_{0}}{1+e^{-(x-x_0)/L_c}}$^64^, $n_c$ being the critical electron density and $L_c$ the characteristic scale length. Such a plasma density profile mimics an exponential ramp^65^ while preserving the total number of plasma particles. For peak plasma electron density $n_{0}\gg n_{c}$, which is usually the case for an overdense plasma, $L_{c} = L\frac{n_{0}}{(n_{0}-n_{c})} \simeq L$. The crucial parameter plasma density gradient scale length *L* can easily be tuned in experiments through the controlled plasma expansion initiated by a prepulse at an adjustable delay and estimated using interferometry^13^. In experiments, such target conditions are easily accessible (for example, the diamond-like carbons with density $\sim2.7\;~{\text{gcm}}^{{ - 3}}$ or thicker carbon foils with density $\sim2.1\;~{\text{gcm}}^{{ - 3}}$ have been previously used^66^).

In a laboratory experiment, the pulse spatial contrast of the focal spot would determine the quality of the transverse plasma profile in the interaction plane. The pulse temporal contrast on the other hand affects the initial longitudinal plasma density profile (its shape and steepness) and thus determines the target conditions at which the main interaction takes place. In order to enable an optimal high harmonic generation process, one needs to ensure a controlled interaction with a predefined preplasma scale length. There are two components to it. Clean interaction is experimentally achieved by employing a main pulse that has a high temporal contrast (high enough so that the pulse pedestal in the interaction region can only reach fluences below the plasma formation threshold of the target material). At the peak intensities utilized for the current investigation, a typical *ps* level pulse temporal contrast of more than $10^{-10}$ would be needed to perform a controlled experiment in the laboratory. This is enabled by the usage of the appropriate laser technology^67^ and/or by improving the available temporal contrast by utilizing the plasma mirror device^68^. The plasma density gradient is controlled by employing a separate pre-pulse for generating a controlled pre-plasma on the target^13^. By varying the delay between the pre-pulse and the high contrast main pulse, the high harmonic generation interaction is optimized^13,28^.

In addition, the PIC simulations performed in this study do not take into account the influence of collisions. The regime of interaction for relativistic high harmonic generation involves ultrashort pulses at relativistic intensity addressing within the pulse duration effects. The typical electron energies involved in the process are such that the cross-sections for collisions are significantly reduced, making such an approximation a reasonably good one. As an illustration, the typical timescale at which electrons with kinetic energy $E_{e}$ and Lorentz factor $\gamma =\left[ E_{e}/m_{e}c^{2} +1 \right]$ collide with plasma ions of charge *Ze* is given by, $\tau _{ie} = \frac{\gamma ^{2}\beta ^{3}}{\Gamma Z n_{e}/n_{c}}$^69^, where $\beta$ is electron velocity in *c*, $\Gamma = 4\pi c n_{c} r_{e}^2 ln\Lambda$, $r_{e}$ is the classical electron radius and $ln\Lambda$ is the Coulomb Logarithm^70^. For the solid target used in our case, $n_{e}/n_{c}\approx 50$ and typical $E_{e}>1~\text{MeV}$, the timescale for collisions $\tau _{ei}$ is more than 1000 times larger than the pulse duration. It has been demonstrated that collisionless PIC simulations successfully address all the aspects of the interaction in this regime^12,21,22,26,27,29,66,69,71^ and hence we have used collisionless, fully relativistic PIC for the investigation. The simulation box of size $\mathrm {30\upmu m \times 30 \upmu m}$ is divided into $8000\times 8000$ cells. Each cell is occupied with 18 electron macro-particles and 3 fully ionized carbon ion macro-particles. The initial electron and ion temperature is set to 100eV. To carry out and complete all the simulation scans, more than 100000 CPU core hours were required in the ELI-ALPS HPC facility.

## Optimal generation of surface high harmonic through plasma tuning

**Figure 1:**
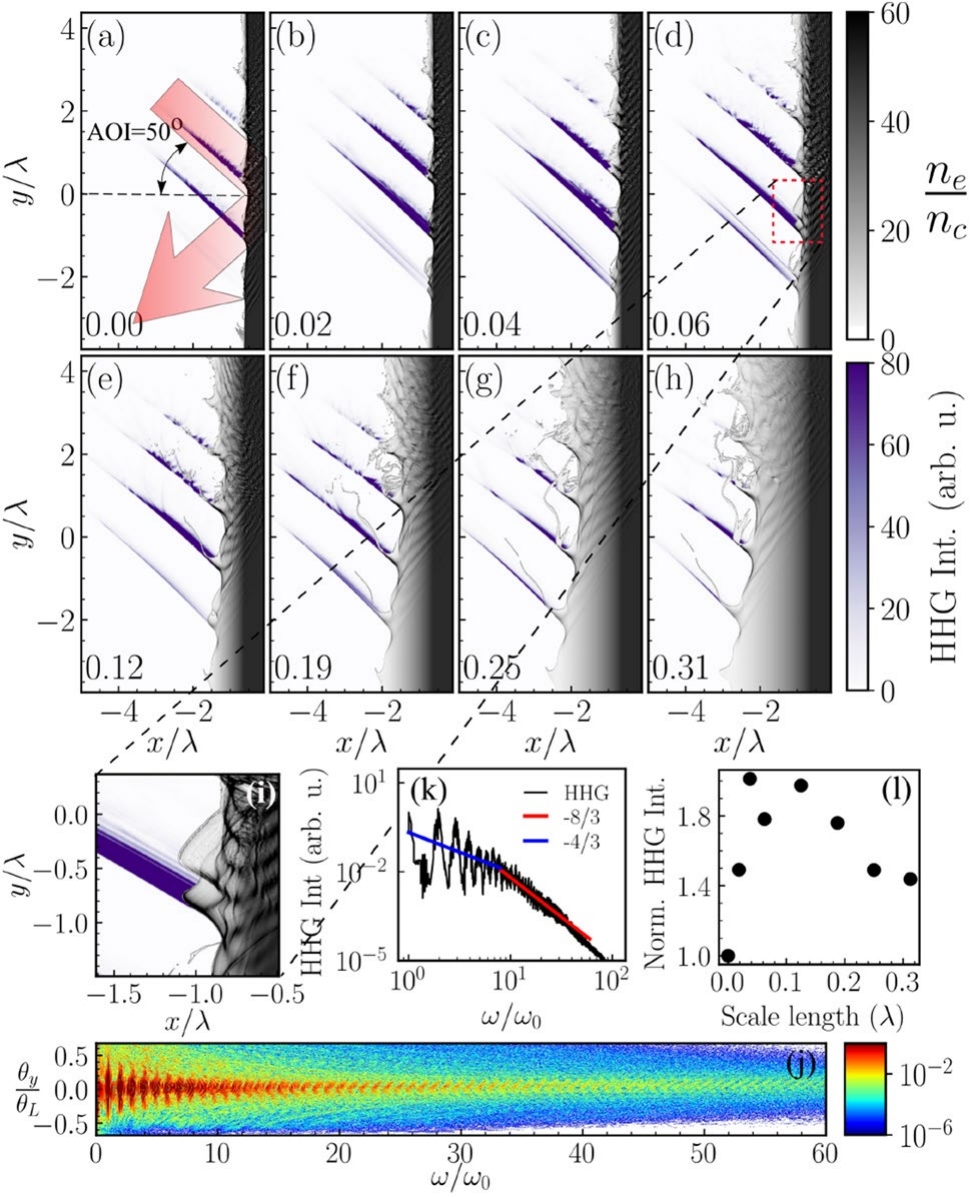
Electron density snapshots during high harmonic generation from a plasma target with plasma density scale-length (L) (**a**) $L=0\lambda$, (**b**) $L=0.02\lambda$, (**c**) $L=0.04\lambda$, (**d**) $L=0.06\lambda$, (**e**) $L=0.12\lambda$, (**f**) $L=0.19\lambda$, (**g**) $L=0.25\lambda$ and (**h**) $L=0.31\lambda$. The numbers in the lower-left corners represent the plasma density scale-lengths (*L*) normalized to the fundamental pulse wavelength $\lambda$. The driving laser light is incident from the top left corner onto the target at 50° to the target normal, and the red shaded arrow in (**a**) indicates the direction of the incident and reflected beam propagation. Attosecond bursts in purple (with wavefront perpendicular to the reflected beam direction) are extracted by applying a Chebyshev type-I band-pass digital filter on the reflected field. (**i**) an enlarged view of the density snapshot shown in (**d**) for a better view of the plasma oscillation at the location of the high harmonic generation. (**j**) High harmonic spectrum generated in 2D-PIC simulation. The colormap represents the harmonic intensity in arbitrary units. The y-axis represents the harmonic emission angle $\theta _{y}$ normalized with laser divergence angle $\theta _L$. (**k**) High harmonic spectrum in log-log scale. The harmonic intensity is normalized to the intensity of the fundamental frequency laser, showing two distinct slopes. Harmonic order 8–60th follows a slope of − 8/3 indicative of harmonics generated via the ROM mechanism. (**l**) Total yield of harmonics 8–60th as a function of the plasma density scale-length (*L*), normalized with respect to the yield at $L=0$.

The high harmonic intensity emitted during the relativistic laser-plasma interaction is optimal for appropriate steepness of the plasma vacuum boundary^13,15,72^. Therefore, a scan on the density scale-length (*L*) is performed in order to investigate the optimum high harmonic generation conditions. Figure 1a–h show electron density snapshots when the peak of the laser pulse interacts with the target having different density gradient scale-lengths, (a) $L=0\lambda$, (b)=$0.02\lambda$, (c) $L=0.04\lambda$, (d) $L=0.06\lambda$, (e) $L=0.12\lambda$, (f) $L=0.19\lambda$, (g) $L=0.25\lambda$ and (h) $L=0.31\lambda$, respectively. Where $\lambda$ is the wavelength of the fundamental frequency driver pulse. The electron density snapshots shown in Fig. 1a–h is normalized to the critical plasma density $n_c$ and is displayed by the gray color-map. Attosecond bursts generated during the interaction are also shown in Fig. 1a–h with a violet color. The colorbar (violet) for attosecond bursts represents the attosecond pulse intensity in arbitrary units.

Figure 1a–h show, three to four attosecond bursts generated during the interaction of steep density plasma with a multi-cycle (pulse-width $\sim$ 8 fs) laser pulse. Despite that electron bunch emission from the target surface is higher for larger plasma density scale-length^61^, the harmonic intensity follows a different trend^13,72^. High harmonic generation is efficient for the sharp density gradient for which mirror-like plasma vacuum boundary is formed and undergoes complex oscillatory motion with relativistic velocity also known as the relativistic oscillatory mirror (ROM) model^52^. Figure 1i shows an enlarged view of Fig. 1d at the location where an attosecond burst begins to arise. The push-pull motion of the ultrashort electron bunch at the plasma vacuum boundary provides all essential oscillatory mirror-like conditions and is responsible for the attosecond burst generation. The high harmonic spectrum is generated by fast Fourier transformation of the electric field snapshot saved at simulation time 73 fs (33 fs after the interaction with the center of the driver pulse), after complete reflection from the plasma mirror. One such harmonic spectrum generated in the interaction with the target with density scale-length $L=0.06\lambda$ is shown in Fig.  1j. The harmonic intensity in arbitrary units is plotted along the color axis, while the y-axis represents the harmonic emission angle normalized with respect to the laser divergence angle $\theta _L$. Figure 1k shows the integrated high harmonic spectrum. The y-axis represents high harmonic intensity normalized to the $\mathrm {1^{st}}$ order harmonic or the fundamental frequency laser intensity. Figure 1k shows that the high harmonic spectrum exhibits two distinct scaling laws. 2nd to 8th order harmonics follow a scaling $I (\omega /\omega _0) \propto (\omega /\omega _0)^{-4/3}$, whereas 8th to 60th order harmonics follow a scaling law $I (\omega /\omega _0) \propto (\omega /\omega _0)^{-8/3}$. The scaling law in high harmonic generation by the relativistic interaction on a plasma mirror is a crucial characteristic which could provide critical information about the interaction regime and dominant mechanisms that are involved in harmonic generation^51^. The scaling law in the high harmonic spectrum shown in Fig.  1k indicates that the dominant mechanism for harmonic generation, in our case, is the relativistically oscillating mirror (ROM). Hence, we consider only from 8th to 60th order harmonics for further investigation. Figure 1l shows the integrated (8th to 60th orders) high harmonic intensity as a function of plasma density scale-length. The y-axis represents high harmonic flux normalized with the high harmonic flux generated from a plasma slab with density scale-length $L=0$. The high harmonic flux sharply increases with density scale-length, by a maximum factor of 2 for $L=0.04\lambda$ and then slowly decreases from $L\ge 0.12\lambda$.

In our coordinate system, the propagation of the reflected and incident electromagnetic fields lie in the *x*–*y* plane, and the propagation coordinate is given by $\eta$ where, $\omega \eta = \left( {\mathbf {r}}\cdot {\mathbf {k}}-\omega t\right) = \omega \left( \frac{{\mathbf {r}}\cdot {\hat{\mathbf {k}}}}{c} - t\right)$ and ${\mathbf {k}}={\hat{\mathbf {k}}}\frac{\omega }{c}$ is the wave vector of the reflected plane wave at $\omega$. The harmonic spectrum presented in Fig. 1j is calculated from the time domain reflected field $E(\eta )$ using, $I(\omega ) \sim |{\tilde{E}}(\omega )|^2 = |FFT_{\eta } \left[ E(\eta )\right] |^2$, where ${\tilde{E}}(\omega )$ represent the reflected electric field in the spectral domain. Attosecond bursts in the time domain, presented in Fig. 1a–h, are calculated from $I_{F}(\eta ) \sim |IFFT_{\omega }\left[ {\tilde{E}}(\omega ).F(\omega _1,\omega _2) \right] |^2$; where *FFT* and *IFFT* refer to Fast Fourier Transform and Inverse Fast Fourier Transform respectively and $F(\omega _1,\omega _2)$ is the band-pass filter function that transmits the frequency band between $\omega _{1}$ and $\omega _{2}$.

## Control of HHG with a two-color ($\omega$–$2\omega$) driving field

The push-pull dynamics on the plasma-vacuum boundary can be tuned to a great extent by engineering the driving field. Subsequently, a considerable control over high harmonic generation can be achieved. Mixing an appropriate amount of second harmonic ($2\omega$) to the fundamental frequency ($\omega$) driving field (two-color field) is a widely used approach to engineer the driving field and has been tested for high harmonic generation from a gaseous medium^73,74^ and bulk semiconductor crystal^75^.

Initial investigations using more tractable 1D PIC simulations hinted at the possibility of enhancing high harmonic emission using multicolor pulses^40,76^. There have been several recent experiments as well as weakly or moderately relativistic laser intensities which indicated that a two-color ultrashort pulse combination can introduce significant modulations in the high harmonic spectral shape^12,41,77^. In this article, we demonstrate that high harmonic generation by relativistic laser-plasma interactions can greatly be optimized by controlling the push-pull dynamics at the plasma vacuum interface using a two-color ($\omega$–$2\omega$) driving field, leading to the emission of isolated attosecond pulses. In order to operate at the near-optimal density scale-length for the high harmonic generation with the fundamental frequency diver, in all two-color ($\omega$–$2\omega$) simulations, we set the density scale-length at $L=0.06\lambda$, .

**Figure 2:**
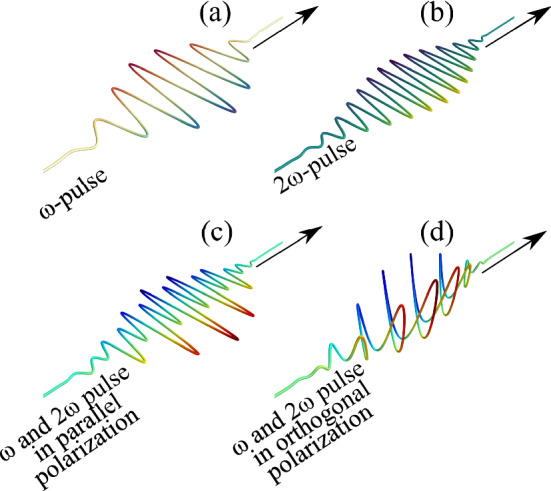
Graphical presentation of the two-pulse configuration which are used in the simulations. (**a**) Representation of pulse with fundamental wavelength (800 nm) pulse. (**b**) Typical second harmonic (2$\omega$) pulse. The electric field of the combined two-color ($\omega -2\omega$) driver (**c**) when $\omega$ and $2\omega$ have parallel polarization, (**d**) when $\omega$ and $2\omega$ have orthogonal polarization with no delay between the two pulses.

A second harmonic ($2\omega$) pulse of wavelength $\lambda _{2\omega } = 400$ nm, pulse-width 8 fs is introduced co-axially with the fundamental frequency ($\omega$) pulse on the plasma target with an identical spot size. The delay between the fundamental frequency ($\omega$) and the second harmonic ($2\omega$) pulses are adjusted in such a manner that positive delay corresponds to a second harmonic pulse arriving later on target. Figure 2a–d graphically presents the two-color driver field configuration that is used in the PIC simulation. Figure 2a,b show the fundamental (800 nm) and the second-harmonic (400 nm) pulses, whereas Fig. 2c,d show the combined optical field when the fundamental and the second harmonic pulses are mixed at an appropriate ratio of vector amplitudes (2:1) at zero time delay and for two different polarization conditions, (c) when both the pulses are in parallel polarization, (d) when the pulses are in orthogonal polarization. We have used two different intensities for the second harmonic ($2\omega$) pulse with $a_{0}^{2\omega }=0.5$ and $a_{0}^{2\omega }=5$ corresponding to laser peak laser intensities $\sim 2\times 10^{18}\, \mathrm {Wcm^{-2}}$ and $\sim 2\times 10^{20}\, \mathrm {Wcm^{-2}}$, respectively, in each configuration.

**Figure 3:**
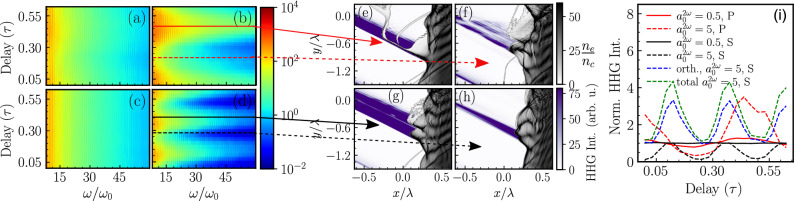
Variation of the high harmonic spatially integrated spectrum with the delay between the $\omega$ and $2\omega$ pulse for fixed $\omega$ and (**a**) $a_{0}^{2\omega }$=0.5 and p-polarized, (**b**) $a_{0}^{2\omega }$=5 and p-polarized,(**c**) $a_{0}^{2\omega }$=0.5 and s-polarized, (**d**) $a_{0}^{2\omega }$=5 and s-polarized, $2\omega$ pulses. The color axis represents the high harmonic intensity in arbitrary units. The delay time is given in units of the fundamental laser time period $\tau =c/\lambda$. (**e**)–(**h**) Enlarged electron density snapshots for the case of high-intensity $2\omega$ pulse and for two different polarizations (s and p-polarization). (**e**) and (**f**) are the electron density snapshots at two important delays between $\omega$ and high-intensity p-polarized $2\omega$ pulse, where both high harmonics fluxes, as well as cutoffs, are maximum (delay $=$ 0.468$\tau$) and minimum (delay= 0.218$\tau$), respectively, as shown by the solid and dotted red lines. In (**g**) and (**h**), we plot the electron density snapshots at two important delays between $\omega$ and high-intensity s-polarized $2\omega$ pulse where both high harmonics fluxes, as well as cutoffs, are maximum (delay $=$ 0.406$\tau$) and minimum (delay= 0.281$\tau$), respectively, and are shown by the solid and dashed black lines. (**i**) High harmonic intensity integrated between 8th and 60th harmonic and normalized with the high harmonic flux generated by the fundamental ($\omega$) driving pulse alone.

In Fig. 3a–d, we plot the high harmonic spectrum as a function of the delay between the fundamental frequency ($\omega$) and the second harmonic ($2\omega$) pulse for four different cases where the second harmonic ($2\omega$) pulse conditions are (a) $a_{0}^{2\omega }$ = 0.5 and p-polarized, (b) $a_0^{2\omega }$ = 5 and p-polarized,(c) $a_{0}^{2\omega }$ = 0.5 and s-polarized, (d) $a_{0}^{2\omega }$ = 5 and s-polarized, respectively. The color axis represents the high harmonic intensity in arbitrary units while the delay is normalized with the temporal period of fundamental frequency (800 nm) pulse, $\tau = c/\lambda$. During the entire simulation, the fundamental frequency ($\omega$) pulse remains unchanged. In Fig. 3a,c, when the second harmonic ($2\omega$) pulse intensity ($a_{0}^{2\omega }$=0.5) is relatively low, the change in the harmonic spectrum is also small. Considerable changes are observed when the second harmonic ($2\omega$) pulse intensity is substantially high ($a_{0}^{2\omega } = 5$) and is shown in Fig.  3b,d. In this case, the high harmonic cutoff frequency as well as high harmonic flux increases and decreases periodically with the delay between the fundamental frequency ($\omega$) and the second harmonic ($2\omega$) pulse. However, the sensitivity is twice for the case of the s-polarized $2\omega$ pulse.

High harmonics are generated by the push-pull-like oscillatory motion of relativistic energy electron bunches at the plasma vacuum interface. The forces that govern the push-pull motion are the radiation pressure by the driving field and the restoring force generated due to charge separation. Typical radiation pressure in relativistic laser solid interaction can be as high as Giga-bar^78^. Therefore, to gain an effective control over the relativistic motion of the electron bunches at the plasma vacuum interface, one would also require a second harmonic ($2\omega$) pulse with strength comparable to that of the fundamental frequency ($\omega$) pulse. Thus, only high amplitude second harmonic ($2\omega$) pulses produce considerable changes in the high harmonic spectrum.

Figure 3e–h show electron density snapshots at the location of the most intense attosecond bursts generation on the plasma vacuum interface for four different conditions, shown by the red (corresponding to high-intensity p-polarized second harmonic pulse) and the black (corresponding to high-intensity s-polarized second harmonic pulse) lines in Fig.  3b,d, respectively. In Fig.  3e–h, the electron density is presented by the gray color-map and HHG flux is presented by the violet color-map. Figure 3e shows the electron density snapshot at the delay $0.468\tau$, where high harmonic flux in addition to the high harmonic cutoff is maximum for the high-intensity p-polarized second harmonic ($2\omega$) pulse and is indicated by the solid red line in Fig. 3b. Figure 3e shows that an intense attosecond burst of XUV radiation is generated when an ultrashort bunch of electrons is ejected from the plasma vacuum interface. Immediately after the ejection, the electron bunch splits into two due to the influence of the two-color driving field. The high-energy part escapes from the plasma surface. However, the low-energy part sharply turns back towards the plasma surface due to the combined field of plasma and laser. The amplitude of the push-pull motion of the surface electron is also maximum at this delay. Similarly, the electron density snapshot at the same location when the high harmonic flux along with high harmonic cutoff is minimum (time delay $= 0.218\tau$) is plotted in Fig.  3f. This configuration is marked with the red-colored dotted line in Fig. 3b. Figure 3f shows that the oscillatory motion of surface electrons is suppressed by the two-color ($\omega -2\omega$) driving field. Consequently, the attosecond pulse intensity is also suppressed. In Fig. 3g, h, we plot electron density snapshots when the high harmonics flux in addition to its cutoff is maximum (delay $=0.406\tau$) and minimum (delay $=0.281\tau$) for the high-intensity s-polarized $2\omega$ pulse. These two configurations are marked with the solid and dotted black lines in Fig.  3d. Figure 3a–h clearly demonstrate that by tuning precisely the delay between the fundamental frequency ($\omega$) and the second-harmonic ($2\omega$) pulse, one can effectively manipulate the electron dynamics at the plasma vacuum interface to manipulate the high harmonic spectrum to a large extent.

In Fig. 3i, we plot the normalized high harmonic flux collected between the 8th and 60th harmonic as a function of the delay between the fundamental frequency ($\omega$) and the second harmonic ($2\omega$) pulse. High harmonic flux is normalized with respect to the high harmonic flux generated from a plane target with a density scale-length L $=$ 0.06 driven by only the fundamental frequency ($\omega$) laser pulse. The red (black) color line represents the high harmonic flux generated with a p-polarized (s-polarized) second harmonic ($2\omega$) pulse. The solid and dashed lines indicate where $2\omega$ intensities are such that $a_{0}^{2\omega }=0.5$ and $a_{0}^{2\omega }=5$, respectively. Marginal enhancement in the high harmonic flux has been observed for low intensity ($a_{0}^{2\omega }=0.5$) p-polarized second harmonic ($2\omega$) pulse at $0.468\tau$ delay. A p-polarized second harmonic ($2\omega$) pulse with higher intensity ($a_{0}^{2\omega }=5$) generates a maximum of 3.5 times enhancement in the high harmonics flux, at $0.468\tau$ delay. For the case of a high-intensity ($a_{0}^{2\omega }=5$) s-polarized second harmonic pulse, the high harmonic flux is suppressed in the parallel direction (along with the p-polarized field) when compared with the harmonic flux generated with only the fundamental frequency ($\omega$) driver pulse. However, a maximum 3.2 times enhancement in high harmonic flux is observed in the orthogonal direction (along with the s-polarized field) which is shown by the dashed blue line in Fig.  3i. The total high harmonic flux (sum of high harmonic flux in parallel and orthogonal directions) is shown by the green line in Fig. 3i. No significant high harmonic flux in the orthogonal direction is observed for the cases shown in Fig. 3a–c.

**Figure 4:**
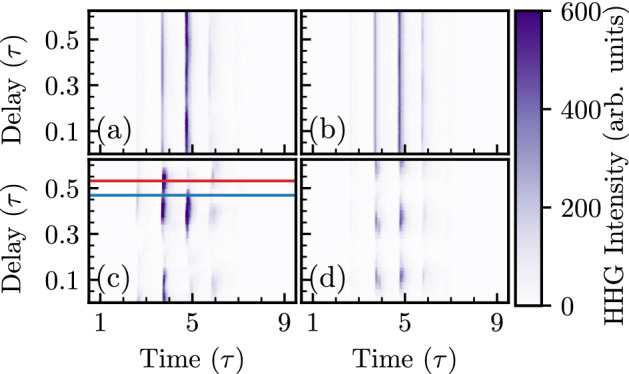
(**a**)–(**d**) Attosecond XUV bursts generated in the interaction for four different configurations of the second harmonic ($2\omega$) pulse, (**a**) $a_{0}^{2\omega }=0.5$, p-polarized, (**b**) $a_{0}^{2\omega }=0.5$, s-polarized, (**c**) $a_{0}^{2\omega }=5$, p-polarized and (**d**) $a_{0}^{2\omega }=5$, s-polarized. The color axis represents the attosecond burst intensity in arbitrary units. A Chebyshev type-I digital filter is applied to obtain contribution between the 8th and the 45th order harmonics.

## Temporal properties of attosecond bursts generated in two-color driving field

Attosecond bursts are time domain representations of high harmonic generation and are generated once in every cycle in relativistic intensity laser interactions with surface plasma^7^. To understand the role of the two-color ($\omega -2\omega$) driving field in attosecond burst generation, one needs to analyze the optical field in the time domain, after completion of laser pulse interaction with the plasma slab. Attosecond bursts are imprinted in the reflected optical field of the driver laser. Filtering out the fundamental ($\omega$) and the second harmonic ($2\omega$) frequency from the optical field results in attosecond bursts in the time domain. In general, in an experiment, spectral selection and dispersion management for *as* pulses can either be done by using transmission-based metal^79–81^ or plasma^82^ filters or reflection-based specially engineered multilayer mirrors^83^. Thus, in a real experimental situation, an appropriate filter can be utilized to optimize the *as* pulse properties.

Figure 4a–d show filtered attosecond bursts as a function of the delay between the fundamental ($\omega$) and the second-harmonic ($2\omega$) pulse for four different second harmonic pulse conditions (a) $a_{0}^{2\omega }=0.5$, p-polarized, (b) $a_{0}^{2\omega }=0.5$, s-polarized, (c) $a_{0}^{2\omega }=5$, p-polarized and (d) $a_{0}^{2\omega }=5$, s-polarized. The attosecond bursts are extracted by applying a Chebyshev type-I band-pass digital filter on the reflected field to extract the contribution of the $\mathrm {8^{th}}$ to $\mathrm {45^{th}}$ order harmonic. In typical experiments, the driving field can be filtered out using an ultra-thin *Al* metal filter of thickness $\mathrm {\sim 50 \, nm}$ without significantly altering the *as* pulse duration. For a typical case, a 50 nm thick aluminium filter transmits approximately from the 10th to the 45th order harmonics^84,85^. In Fig.  4d, we plot attosecond bursts in the orthogonal direction as the high harmonic flux is predominantly generated in the orthogonal direction.

The modulation of the attosecond burst intensity with the delay between the fundamental ($\omega$) and the second-harmonic ($2\omega$) pulse is consistent with the high harmonic flux plotted in Fig. 3i. As expected, significant modulation in the attosecond burst intensity with the delay has been observed when the second-harmonic pulse intensity is significantly high (Fig. 4c,d). Further investigation reveals an interesting aspect of the two-color ($\omega -2\omega$) field-driven high harmonic generation. The modulation in the driver field due to the second harmonic pulse can suppress several attosecond bursts while allowing only one, leading to the efficient generation of an isolated attosecond pulse.

**Figure 5:**
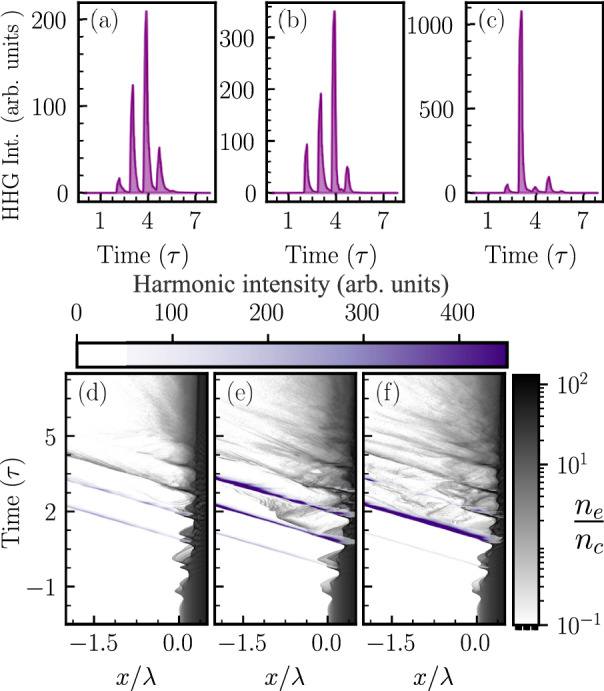
Attosecond bursts intensity for harmonics 10–45 generated (**a**) by the fundamental frequency driving pulse and (**b**), (**c**) by the two-color driving pulse; (**b**) at delay $0.468\tau$, (**c**) at delay $0.531\tau$ for the case of high-intensity ($a_{0}^{2\omega }=5$) p-polarized second harmonic ($2\omega$) pulse. (**d**)–(**f**) Space-time dynamics of the surface electron density and attosecond bursts for the three different conditions given above and for which the attosecond bursts are plotted in (**a**)–(**c**), respectively.

In order to examine conditions for isolated attosecond pulse generation and compare them with attosecond bursts generated by only the fundamental frequency ($\omega$) driving pulse, we have plotted the attosecond burst intensity in the time domain for three different conditions in Fig. 5a–c. Figure 5a shows attosecond bursts generated by the fundamental frequency driving pulse. Figure 5b,c show attosecond bursts generated when the delay between the fundamental frequency ($\omega$) and the second-harmonic ($2\omega$) pulse is $0.468\tau$ (marked with the blue line in Fig.  4c), when the high harmonic intensity is maximum, and at delay $0.531\tau$ (marked with the red line in Fig. 4c), when an isolated attosecond pulse is generated with the highest contrast. Isolated attosecond pulses with lower contrast are also generated at delays $0.031\tau$, $0.125\tau$, and $0.343 \tau$. Figure 5a shows that nearly four attosecond bursts are generated when the surface plasma is driven by only the fundamental frequency laser pulse. Only one of the attosecond bursts is intensified, while all other attosecond bursts get suppressed at a delay $0.531\tau$ by the two-color driving field. Figure 5a–c clearly demonstrate that by tuning the delay between the fundamental and the second-harmonic pulse, one can effectively control the attosecond burst distribution in time domain.

The interpretation of attosecond bursts management, mentioned above, is associated with the behavior of the electron density for the particular driver pulse configuration. Figure 5d–f show the space-time dynamics of the electron macro-particle density generated using two-dimensional PIC simulation results by scanning through the time series of electron density snapshots. The electron density normalized to the critical electron density is presented by the gray color-map. The violet color-map represents the attosecond burst’s intensity in arbitrary units. Attosecond bursts are generated at each spike in the space-time electron density during the push-pull dynamics of the surface electrons which are responsible for the attosecond bursts generation and is strongly modified in the two-color driving field resulting in the control of attosecond bursts in the time domain. Comparing Fig. 5d,e, the amplitude of electron spikes is larger for all attosecond bursts at a delay $0.468\tau$ which caused 3.4 times enhancement in the high harmonic flux (Fig. 3i). In Fig.  5f, the first electron spike amplitude decreases, resulting in a decrease in the intensity of the first attosecond burst. The amplitude of the electron spikes increases substantially for the second attosecond bursts. Consequently, the intensity of the second attosecond burst enhances drastically. The next two electron spikes spread and are nearly eliminated by the two-color field. Consequently, the attosecond bursts are also weak in intensity. Hence, at $0.531\tau$ delay, an intense isolated attosecond pulse is generated with temporal width (FWHM) of approximately 200 *as*.

We would note that the XUV *as* pulses are predominantly emitted in each drive laser cycle by one primary bunch. The spectral extent of the sub-cycle emission is linked with the acceleration of the bunch in the combined field and scales up with the average Lorentz factor of the bunch. The emitted power of the coherent emission scales with bunch electron density $n_{b}^2$ at the time of emission. The electron bunch, during the interaction, undergoes complex spatio-temporal dynamics in our case, under the influence of: (a) the incident driving ultrashort two-color fields; (b) the ensuing plasma charge separation field, and (c) the reflected multicolor electric fields. The complete process is strongly dependent on the polarization of the incident and reflected electric fields and is significantly linked to the geometry of the interaction, like the angle of incidence and focal spot size. In addition, it is also completely dependent on the plasma properties like $L/\lambda$. In addition, during the interaction, when the *as* pulse train is emitted from the relativistic plasma mirror (PM), the PM undergoes denting^22^ which leads to the sharpening of the PM density gradient due to laser radiation pressure. Such a process introduces changes in the instantaneous plasma density gradient seen by the different cycles of the driver laser during the interaction with the PM. This affects the formation and the dynamics of the electron bunches and consequently impacts the *as* pulse train emission efficiency throughout the pulse duration. At high intensities where denting within the pulse duration is relevant^22^, this indeed leads to suppression of harmonic emission efficiency during the second half of the pulse, which has been confirmed previously in 3D PIC simulations^35^. All these factors play a role in deciding the optimal emission condition. The interaction simultaneously depends on multiple parameters in an interconnected fashion. This makes the optimization approach based on tuning one control parameter at a time much more difficult, and sometimes even impossible. But, recent studies indicate that there is significant scope to optimize the properties of the *as* pulses described here by adopting more advanced techniques based on machine learning^86,87^.

## Conclusions and outlook

In this work, we have shown that by tailoring the temporal shape of the interacting laser field, it is possible to generate isolated attosecond pulses all optically. The results show that a fourfold enhancement in the high harmonic flux can be achieved by mixing an appropriate amount of the second harmonic with the fundamental frequency driving pulse and precisely controlling the delay between the two fields. An optimal two-color driving field favourably modifies the spatio-temporal dynamics of the relativistic surface electrons, enabling one to control the attosecond burst generation in the time domain. Under such optimal conditions, an isolated attosecond burst of duration 200 *as* (FWHM) can be generated. The laser-plasma parameters presented and investigated in our work are accessible by the currently available state-of-the-art lasers and target designs. The results are valid for a few-cycle, carrier-envelope phase stabilized, high temporal contrast, high repetition rate relativistic intensity lasers^88,89^, and the predictions can be scaled up to the interaction driven by high-intensity low repetition rate Petawatt class lasers available worldwide^90^. In several investigations the efficacy of the high-intensity plasma medium in controlling the recombination of laser beams^91^, tight focusing of both the laser^92,93^ and generated high harmonics^22^ and diffraction both static^71,94^ as well as dynamic diffraction^26^ of the generated harmonic pulses have already been experimentally demonstrated. Temporal light field shaping in combination with such existing plasma optics technologies has the potential to provide a viable route to achieve very high fields in the XUV or X-rays, opening up the possibility to study non-linear XUV processes in atomic and molecular physics^95^ or even to pursue extreme high field physics circumventing the limitations of the laser technologies^29^.

## Data Availability

The data used for preparing the figures in the current study could be available from the corresponding authors upon reasonable request.
